# The Use of Systematic Reviews and Other Research Evidence in Disasters and Related Areas: Preliminary Report of a Needs Assessment Survey

**DOI:** 10.1371/currents.dis.ed42382881b3bf79478ad503be4693ea

**Published:** 2013-01-22

**Authors:** Bonnix Kayabu, Mike Clarke

**Affiliations:** Evidence Aid Coordinator, Centre for Global Health, Trinity College Dublin, Ireland; Director, All Ireland Hub for Trials Methodology Research; Evidence Aid

## Abstract

Objectives: This paper presents the initial data analysis for a survey to identify the attitudes towards systematic reviews and research of those involved in the humanitarian response to natural disasters and other crises; their priorities for evidence, and their preferences for accessing this information. Methods: Snowballing sampling techniques were used to recruit participants who identified themselves as humanitarian aid workers, with or without experience in providing funding to aid agencies. An online questionnaire with both quantitative and qualitative questions was made available to participants using a variety of e-mail lists. Quantitative responses from 85 participants to a selection of questions were descriptively analysed using SPSS. Results: Findings indicated that respondents had positive opinions about systematic reviews and using research evidence when planning and responding to disasters. Seventy participants answered the question on the usefulness of reviews before, during and after disasters and, of these, 83% said that systematic reviews are useful in disasters, and the remaining 17% said they did not know. No-one selected the option that systematic reviews are not useful. The most preferred format for access to systematic reviews was the whole reviews, supplemented by comments from experts in the humanitarian sector (61%), 33% choose access to the full review, 20% choose the summary of reviews and 50% choose summary of reviews plus context-specific information. Inadequate access was the most commonly reported barrier to the use of systematic reviews (70%). This was followed by the lack of time to use reviews (59%) and insufficient knowledge about reviews (49%). Respondents selected scientific evidence as the most preferred type of evidence for influencing their decisions (80%), 11% ranked personal experience highest, 6% said their organisation’s usual practice, 1% said anecdotal evidence and 1% said intuition would be their first choice. 69% of participants “strongly agreed” that evidence from systematic reviews could have a positive role in humanitarian interventions and a further 29% “agreed” with the same statement. 66% thought they would like to access them when a natural disaster is not known to be imminent, compared to 34% who said that they would not wish to access systematic reviews at such a time. 70% would like to access systematic reviews during the period of prediction that a disaster will happen Conclusion: These preliminary findings from the Evidence Aid survey emphasise the need for “global” evidence but also the need that this be supplemented by local and context-specific knowledge. Systematic reviews could play a central role in improving the effectiveness of humanitarian aid in the planning, delivery and recovery phases of a disaster. Keywords: Evidence base, humanitarian planning, delivery and recovery, systematic review

## BACKGROUND

This paper presents the initial data analysis for a survey to identify the attitudes towards systematic reviews and research of those involved in the humanitarian response to natural disasters and other crises; their priorities for evidence, and their preferences for accessing this information. The survey is being conducted by Evidence Aid and the instrument is available in Arabic, English, French and Spanish (www.EvidenceAid.org). It remains open to anyone involved in the disaster field, or working in humanitarian relief more generally and has been published 1 .

The role of systematic reviews in health 2 ^,^ 3 and other areas 4 ^,^ 5 is increasingly well established. However, there are substantial gaps between the need for systematic reviews and the existence of up-to-date reviews. For instance, 200 areas of uncertainty about the effects of interventions were identified in the months after the Indian Ocean tsunami of December 2004, systematic reviews existed to help people to make decisions about the implementation of less than one quarter of these interventions 6 . Since then, the number of relevant reviews has increased but there is an ongoing need to increase further the production of systematic reviews that will be relevant in the humanitarian sector, and to facilitate the conduct and availability of the research that forms the raw material for these reviews 7.

Evidence Aid is an international initiative which is working together with its partners to improve access to systematic reviews of relevance to disasters. This survey is another step in its efforts to identify attitudes towards systematic reviews, and other forms of research, among those involved in the humanitarian response to natural disasters and other crises; their priorities for evidence, and their preferences for accessing this information. The findings will help Evidence Aid to develop the means to make it easier for humanitarian assistance stakeholders to access up-to-date knowledge that will help them make often difficult choices about interventions and actions.

Systematic reviews are key to well-informed decision-making in many areas. They can be used to inform decisions where the aim is to increase the impact and the value of money 8 , and they make it easier for people to overcome the problem of an ever-increasing volume of information available in both on online databases and in printed material, by providing summaries of research relevant to a particular topic 3 . Importantly, they can minimise the impact of bias, by promoting transparency of methods and detailed, structured reporting, and avoiding undue emphasis on individual studies 9 ^,^ 10 .

A significant body of knowledge has been accumulated about public health interventions in emergencies and there have been calls for the development of an evidence-base of humanitarian health interventions 11 . A recent post-2015 framework for Disaster Risk Reduction issued by the UNISDR recognises that there is a significant amount of information on what good practice is in disaster risk management and what works, but identifies challenges in accessing and using this information. It highlights the need to develop and provide more guidance, principles and tools on how good practice is achieved 12 .

The survey aims to identify the challenges that aid agencies face when they need up-to-date knowledge to plan and respond to natural disasters and other humanitarian emergencies 13 . It also aims to establish the perceived importance of systematic reviews in disaster settings and to identify the preferences of aid workers for ways to access knowledge. This initial report focuses on the findings of the survey for these high-level objectives (Table 1). A future, fuller analysis when more responses have been received will deal with the survey as a whole.

**Table 1. Analysed questions from the full Evidence Survey:** 

2.6 If you needed to access the findings of systematic reviews, how would you like them to be presented to you?
2.7 If you needed to access the findings of systematic reviews, how would you like to do this?
2.8 Have you used systematic reviews as a source of evidence in decision­making?
2.11 How useful do you think systematic reviews can be before, during and after disasters?
2.15 Rank these different types of evidence that might influence your decisions (Anecdotal evidence; Intuition; Personal experience; Scientific evidence; Culture norms; Organizational usual practice).
2.18 When would you like the evidence from systematic reviews to be presented to you?
2.19 Would the findings of systematic reviews affect the implementation of your interventions?
2.22 Do you think that improved access to systematic reviews could play a role in improving the response to disasters?
2.24 What could be the barriers to your use of systematic reviews?
2.26 Please indicate your response to the following statements (Evidence from systematic reviews could have a positive role in humanitarian interventions; Humanitarian interventions should be based on reliable knowledge of which interventions work, which interventions don’t work, and which ones are harmful; The use of evidence from systematic reviews will make humanitarian interventions more cost-effective; Systematic reviews are for academics, not for humanitarian workers; Evidence from systematic reviews is only useful for humanitarian interventions in health care; Evidence from systematic reviews is useful for humanitarian interventions in areas other than health care; Humanitarian workers are so busy during a natural disaster that searching for evidence is not possible; Systematic reviews devalue unpublished literature, such as internal reports of agencies; Systematic reviews devalue the experience of humanitarian workers; Evidence from systematic reviews is not practical for making decisions about humanitarian intervention; Systematic reviews should give alternative options when the best option is not available or cannot be used for a specific decision about humanitarian interventions)
2.33 What attitudes do you think donors have towards systematic reviews?
3.12 Do you think the use of systematic reviews can help you to assess the likely effects of projects before providing funding to agencies?
3.13 Do you use systematic reviews to assess the likely impact of projects before providing funding to agencies?

## METHODOLOGY

The design of the Evidence Aid survey has been reported previously 1 and is summarised here. It was developed following a formal evaluation of Evidence Aid in 2008/09 14 and subsequent discussions with people working in a variety of aid agencies, covering health, nutrition, water and sanitation, and displaced people. This preparatory work helped to raise awareness of Evidence Aid, allowed a discussion of the role of research evidence in decision-making, and helped with the selection of questions for the survey.

A small group of people piloted the survey, leading to further changes and refinements to the online English version, before its launch in July 2011. The English version was translated into Arabic, French and Spanish - recognising the potential language barrier for many people and organisations involved in disasters or humanitarian relief and with the intention of obtaining rich information from aid workers in different regions of the world, whose culture and aid agencies’ agendas might differ markedly from those of Anglophone organisations.

The survey assesses the potential role of systematic reviews using mixed methods, with a combination of quantitative and qualitative questions. This report focuses on some of the quantitative data.

Information about the survey was sent to contacts established during the aforementioned discussions, and a snowballing technique was used to cascade it to others. This included distribution through the Information Services of the World Health Organisation, World Association for Disasters and Medicine (WADEM), Trinity International Development Initiative (TIDI), the email distribution list of the US Center for Disease Control and Prevention (CDC) and participants at the Measurement of Specific Sphere Indicators (MeSSI) meeting in Atlanta in February 2012. The survey was also distributed to participants in the Médecins Sans Frontières Scientific Day 2012 and posted on the Active Learning Network for Accountability and Performance in Humanitarian Action (ALNAP). All respondents were encouraged to circulate the survey to others.

The survey is available online in Survey Monkey and responses were transferred into an Excel spreadsheet for these analyses. After data cleaning, the data were transferred into SPSS 18.0 for a second data cleaning; followed by a descriptive data analysis. These preliminary analyses includes responses from the first 80 participants who answered the English version of the survey and five participants who responded to the French version. Responses to the Arabic and Spanish versions will be considered during the future, comprehensive data analysis.

## RESULTS

**Demographics**

The 85 respondents to the online survey hold a variety of academic qualifications including PhD degrees (17 people), Medical degrees (24), MPH (32), MSc (16), MA (13), and MBA (6). Half were located in either Western Europe (28%) or North America (25%). Others were located in Sub-Saharan Africa (13%), Asia (13%), Australia or New Zealand (8%), Middle East (6%), Eastern Europe (2%) and South America (2%). The respondents cover a wide range of experience of working in the humanitarian sector: 25% had 1-5 years experience, 19% had 5-10 years, 12% had 20-25 years, 6% had 30-35 years and 3% had 35-40 years. Respondents have worked in natural disasters, conflict settings and other types of disasters, with many working in both conflict and natural disaster settings (40%), and 8% working only in natural disasters setting. 3.5% of respondents have worked in other types of disasters.

**Use of systematic reviews in disasters**

Seventy participants answered the question on the usefulness of reviews before, during and after disasters. Of these, 83% said that systematic reviews are useful in disasters, 17% said they did not know and no-one selected the option that systematic reviews are not useful. Of the 70 participants who answered the question about their experience in using systematic reviews, more that half (53%) had experience of using systematic reviews in decision making.

**Format of systematic reviews to be accessed**

Participants were asked how they would like to access findings of systematic reviews, and could select more than one option. All 85 respondents answered this question and revealed a preference for access to whole reviews supplemented by comments from experts in the humanitarian sector (61%), 33% choose access to the full review, 20% choose the summary of reviews option and 50% choose summary of reviews plus context-specific information. There was also support for the addition of local information to systematic reviews: 47% of participants said that this should always be available, 50% said sometimes and 3% said rarely.

**Barriers to the use of systematic reviews**

Inadequate access was the most commonly reported barrier to the use of systematic reviews (70%). This was followed by the lack of time to use reviews (59%) and insufficient knowledge about reviews (49%). A high proportion of respondents (82%) felt that improved access to systematic reviews would improve responses to natural disasters and other humanitarian crises, with 18% reporting that they were not sure. No-one chose the option that improved access to systematic reviews would not improve humanitarian assistance.

In considering other challenges that could make it difficult to use systematic reviews in disasters and other humanitarian crises, one respondent (2%) said systematic reviews would make planning and delivery of services difficult, but 26% said that the academic language used in systematic reviews is difficult to understand and 11% gave other barriers to the use of reviews.

**Methods of access to systematic reviews**

Online access to systematic reviews was the most preferred way to access reviews (83%), followed by access via email and access on CD or DVD (18%). Despite the recent increase in popularity of mobile technology, 28% of respondents said access to full systematic reviews via mobile technology was acceptable but not preferred, and only 5% said it was preferred. On the other hand, 33% said access to summaries of systematic reviews via mobile technology was acceptable but not preferred, and 12% said it was preferred.

**Types of evidence likely to influence decisions**

Participants were asked to rank which of the following six types of evidence were most likely to influence their decisions in disasters: anecdotal evidence, intuition, personal experience, scientific evidence, culture norms and organizational usual practice. Scientific evidence was the most preferred (80%), 11% ranked personal experience highest, while 6% said their organisation’s usual practice, 1% said anecdotal evidence and 1% said intuition would be the first choice. Alongside the 8 respondents who gave personal experience as their first choice, it was the second choice for 22 respondents and the third choice for a further 22. Anecdotal evidence, on the other hand, was ranked as the least important by 27 respondents and was the first choice for one and second choice for four respondents. Similarly, intuition was among the least likely type of evidence to be chosen. It was selected as the least important source of evidence by 20 respondents and selected as the most important source of evidence by one person.

**Timing for the use of evidence from systematic reviews**

On the question of when participants would like to access finding from systematic reviews, 66% thought they would like to access them when a natural disaster is not known to be imminent, compared to 34% who said that they would not wish to access them at that time. 70% would like to access systematic reviews during the period of prediction that a disaster will happen (e.g. when a hurricane is known to be heading for an area), 51% during and shortly after disasters, and 56% after disaster (i.e. during the period of recovery and development work). Respondents were able to select more than one answer to this question and the relevant data are shown in Table 2. This shows that the largest single group, about a quarter of the respondents (n=22, 27%) thought they would like to access systematic reviews at all four time points.

**Table 2. Preferences for timing of use of systematic reviews:** Choices: A: When a natural disaster is not known to be imminent; B: During the period of prediction that a disaster will happen (ie when planning the response to a specific type of disaster which is likely to strike in the near future, such as a hurricane); C: During and shortly after a disaster; D: After a natural disaster (i.e. when evaluating the response to a specific disaster)

Choices	Number of people	%
A B C D ABCD AB BC ABC ABD AD BCD CD AC	46 49 36 39 22 5 4 3 3 3 1 1 1	66% 70% 51% 56% 27 6 5 4 4 4 1 1 1

**Potential role of systematic reviews**

Overall, opinions on the potential role of systematic reviews were positive: 69% of participants “strongly agreed” that evidence from systematic reviews could have a positive role in humanitarian interventions and a further 29% “agreed” with this statement. Most respondents thought that humanitarian interventions should be based on reliable knowledge of which interventions work, which interventions don’t work, and which ones are harmful; with 26% “agreeing” and 71% “strongly agreeing” with the statement. More than half the respondents thought that the use of systematic reviews will make humanitarian interventions more cost-effective (41% strongly agreed, 29% agreed and 6% disagreed) and most (55%) disagreed that systematic reviews could devalue experience of humanitarian workers.

The majority of respondents disagreed with statements about systematic reviews being for academics and not for humanitarian aid workers (50% “disagreed” and 30% “strongly disagreed”), with few in favour of this statement (6% “agreed” and 3% “strongly agreed”). The respondents also disagreed that evidence from systematic reviews is limited to evidence for humanitarian health interventions (51% disagreed and 37% strongly disagreed), suggesting support for systematic reviews in choices about issues such as shelter, education, security, etc. Most respondents did not agree with the suggestion that evidence from systematic reviews is not practical for making decisions about humanitarian interventions (50% “disagreed” and 20% “strongly disagreed”, while 16% “agreed” with this statement).

**Donor agencies and systematic reviews**

In response to the question about whether donors do, or do not, encourage the use of evidence from systematic reviews, no respondent thought that they were opposed to their use. Whereas, 30% thought that they neither encourage nor discourage their use, 25% thought that donors are not aware of systematic reviews and 31% thought they are interested in their use.

The 25 respondents who had experience in working with agencies that fund humanitarian relief thought that systematic reviews could help to assess the effects of projects before delivery of funding to them (83% said Yes, 13% said No and 4% said they don’t know). Also, 54% of these 25 respondents thought that systematic reviews could help to assess the impact of projects.

## DISCUSSION

Many aid workers recognise the increasing need to promote best practice in disasters and the value of using scientific evidence when planning and responding to disasters and making choices about interventions and actions. However, comprehensive reliable and up-to-date evidence on which interventions work (in what setting, under what conditions), which don’t work, which might be harmful and which are unproven is still lacking. Our discussions with some humanitarian stakeholders when planning this survey revealed the poor quality of the evidence, the tools used to measure the effects of humanitarian assistance, and the data in some disaster databases 15 ; as illustrated by the following quotes.

“ Evidence-based practice in humanitarian response is very poor. We do most things because we have been doing them year after year, we don’t do them because we have proven they are right. Very few have been really proven…”

Further revision of the Sphere Handbook 16, principally through a set of guidelines that are set out in the Humanitarian Charter and Minimum Standards in Disaster Response) should be welcomed by the humanitarian stakeholders, The revision of the Handbook seeks to define and uphold the standards by which the global community responds to the plight of people affected by disasters and should ensure that the Handbook provides information with a robust and reliable scientific base. The revision will also help in the choice of indicators and the way they can be measured in the field, helping to resolve a challenge highlighted by a participant in the discussion to develop the Evidence Aid survey:

“ … *Most of the indicators are not measurable. And it doesn’t tell you how to measure them either. Even for the few, which are measurable, it doesn’t give any methodology…”*

Initiatives such as MeSSI (Measurement of Specific Sphere Indicators) are addressing the quality of indicators used in disasters. The CDC are collaborating with Columbia University in the USA to take a leading role in this work and an expert meeting was held at CDC in Atlanta in February 2012, with a delegate from the Sphere Project. This meeting focused on the selection and the measurement of the so-called “Sphere indicators”.

During our discussions with aid workers while preparing this survey, the need for users of disaster databases to be cautious and more analytical about the quality and accuracy of data in these databases was also highlighted.

Finally, reporting mechanisms within the humanitarian sector were identified to be a major concern. People who write reports for non-Governmental Organisations (NGOs) are not necessarily familiar with how the source data were collected in the field. This has a potential to produce interpretations of those data that have little to do with the true situation. Furthermore, although the narrative of the reports might allow an NGO to claim “international standards were met” and that their interventions and actions are effective, poor quality data could undermine the reliability of such conclusions. An additional problem is that it can be difficult to separate the contribution of the local disaster management team and of other humanitarian stakeholders in such reports. One respondent said:

“*It is important to start analysing the effectiveness of interventions, it is challenging but possible. The problem with aid in general is that it is made by a lot of anecdotal reports. There are more opinions than really hard data in NGO reports…*”

The responses to the Evidence Aid survey reveal the perceived usefulness of, and demand for, systematic reviews amongst workers and agencies that respond to natural disasters and other humanitarian crises. Many respondents would like access to full reviews, combined with advice from humanitarian experts and local information. This is in keeping with the fact that the researchers who prepare and maintain systematic reviews are unlikely to be experts in disasters and the reviews are unlikely to be focused on the effects of interventions or actions in disaster settings. These expert commentaries could make it easier for users to understand the applicability of the findings of the reviews and to decide on the implementation of interventions and actions.

The preliminary findings presented in this paper emphasise the need for “global” evidence but also the need that this be supplemented by local and context-specific knowledge. They also highlight the need for knowledge producers to interact with potential users to help identify key priority questions and to have effective communication strategies to share this information with humanitarian aid workers.

## LIMITATIONS OF THE STUDY

Our findings reflect the views of the 85 participants, almost all of whom use the English language, and might not be representative of the wider humanitarian community. More responses and the addition of findings from the Arabic and Spanish versions could provide important additional information. Another potential limitation of this study is that some of the respondents were closely linked and could share similar views. The views of the participants have not yet been tested against other sources and the additional comments from respondents did not provide sufficient information to allow us to dig deeply into the respondents’ views. Further research will investigate how various confounding factors are related and how they might influence the attitudes of aid workers to systematic reviews.

Despite these limitations, this study is the first of its kind, introducing many humanitarian stakeholders to systematic reviews and their potential role in helping those who are responsible for delivering humanitarian relief.

## CONCLUSIONS

During humanitarian response, the main focus for aid workers is the provision of support to people affected by the disaster. However, this should not mean that they are unable to make use of evidence when making decisions about interventions and actions, especially if such knowledge exists and can be accessed. The preliminary findings of the survey reported here support this view and identify both a need for evidence and a recognition of this need. Priorities for different types of evidence will be examined during the comprehensive analysis of the survey data, and through future interviews with key informants.
